# Genotype-environment interaction on human cognitive function conditioned on the status of breastfeeding and maternal smoking around birth

**DOI:** 10.1038/s41598-017-06214-y

**Published:** 2017-07-20

**Authors:** S. Hong Lee, W. M. Shalanee P. Weerasinghe, Julius H. J. van der Werf

**Affiliations:** School of Environmental and Rural Science, University of New England, Armidale, New South Wales 2351 Australia

## Abstract

We estimated genotype by environment interaction (G × E) on later cognitive performance and educational attainment across four unique environments, i.e. 1) breastfed without maternal smoking, 2) breastfed with maternal smoking, 3) non-breastfed without maternal smoking and 4) non-breastfed with maternal smoking, using a novel design and statistical approach that was facilitated by the availability of datasets with the genome-wide single nucleotide polymorphisms (SNPs). There was significant G × E for both fluid intelligence (p-value = 1.0E-03) and educational attainment (p-value = 8.3E-05) when comparing genetic effects in the group of individuals who were breastfed without maternal smoking with those not breastfed without maternal smoking. There was also significant G × E for fluid intelligence (p-value = 3.9E-05) when comparing the group of individuals who were breastfed with maternal smoking with those not breastfed without maternal smoking. Genome-wide significant SNPs were different between different environmental groups. Genomic prediction accuracies were significantly higher when using the target and discovery sample from the same environmental group than when using those from the different environmental groups. This finding demonstrates G × E has important implications for future studies on the genetic architecture, genome-wide association studies and genomic predictions.

## Introduction

There has been considerable interest in effects of breastfeeding and maternal smoking around birth on cognitive function and later performance such as intelligence, memory and educational attainment. Many studies have suggested breastfeeding influences cognitive function and intelligence^1–3^, e.g. breastfeeding was associated with higher cognitive development than was formula feeding. On the other hand, there are reports that breastfeeding has little effect on intelligence in children after stringent correction for socio-demographic factors^4, 5^. There are also a number of studies reporting that maternal smoking around birth has negative effects on intelligence and later performance^6–9^ whereas some studies show no such effects after adjusting for confounding effects such as socio economic status^10, 11^. There is an interesting study by Batstra *et al*. (2003) demonstrating that the adverse effects of maternal smoking on children’s cognitive functions and performance were limited to those who had not been breastfed^12^, which is one of few studies accounting for the combined effects of breastfeeding and maternal smoking around birth. However, there are no studies into a genotype by environment interaction (G × E) for later cognitive performance conditional on the maternal environment as determined by breastfeeding or/and maternal smoking around birth. Hence, it is unknown whether the genetic expression for these traits differs between these maternal environments.

Genes have the ability to react and produce alternative phenotypes in response to the environment. Genetic variation in response to environmental conditions has been described as phenotypic plasticity, reaction norms or G × E. The phenomenon is widespread and one of the fundamental factors in biology and evolution^13–16^. G × E can be estimated in an experiment where individual or relatives’ phenotypes can be measured in different environments. However, measurement of relatives across environments is not feasible in human populations as family sizes are small and often confounded with environments and limited data exists on genetically related individuals measured across environments. The limited focus on G × E in human genetic studies is therefore unsurprising. However, when using genomic information it becomes much easier to obtain information about the same genotypes measured in different environments. Therefore, genomic data provides opportunities to estimate G × E in human data.

In this study, we estimate G × E on later cognitive performance and educational attainment across the maternal environments for breastfeeding and smoking using a novel statistical approach that is facilitated by the availability of datasets with the genome-wide single nucleotide polymorphisms (SNPs). The use of genome-wide SNP data on unrelated individuals to determine genotype effects across environments presents a paradigm-shifting approach to dissect the genetic architecture of complex traits^17–20^. In the approach, two individuals who are not related in the conventional sense can be compared experimentally, because they share part of their genome by descent and this information can be derived from genome wide SNP genotypes. Since the unrelated individuals do not share common environments, any covariance between their shared genome and their phenotype is most likely genetic and not environmental. Genetic data create links between individuals in the population so that the estimation of G × E and testing of related hypotheses do not require measures of relatives in different environments, or longitudinal data on the same individuals. The proposed approach can be applied whenever phenotypes are recorded and genotype data are collected across known environmental conditions, in this case, breastfeeding and maternal smoking status. We use a multivariate linear mixed model^21, 22^ to estimate genetic variance and covariance based on relatedness derived from genome-wide SNP genotypes. A genetic correlation (i.e. scaled genetic covariance) between the phenotypic expression of genotypes in different environments, which is significantly different from 1, indicates evidence of G × E^23, 24^.

## Results

We used the UK Biobank database (http://www.ukbiobank.ac.uk)^25^ where there were genotyped individuals measured for cognitive traits and environmental variables (see Methods). We preliminarily analysed the phenotypic data using a multi-trait genomic residual maximum likelihood (GREML) to dissect shared genetic architecture between the cognitive traits. Then, it was primarily focused to estimate G × E explained by the genome-wide SNPs using a whole genome approach. In the approach, we used a multi-variate GREML to estimate genetic variance and covariance explained by the genome-wide SNPs for each cognitive trait across different environmental conditions of breastfeeding and maternal smoking status (see Methods). We carried out the analyses on the four unique environmental groups, i.e. 1) breastfed without maternal smoking, 2) breastfed with maternal smoking, 3) non-breastfed without maternal smoking and 4) non-breastfed with maternal smoking. The rationale for such grouping is that the environmental effects of breastfeeding and maternal smoking are not likely to be additive^12^. Therefore, the combination of breastfeeding and maternal smoking status would generate four distinct environments that could be used to test our hypothesis with a novel approach to estimate G × E.

After data quality control (QC), a sample of 25,445, 78,283, 77,919 and 77,750 genotyped and phenotyped individuals was available for respective analysis of fluid intelligence, memory, reaction time and educational attainment. The distribution of samples classified by breastfeeding and maternal smoking status is shown in Table 1.

**Table 1:** The number of samples for the status of breastfeeding and maternal smoking around birth.

	Fluid intelligence	Memory	Reaction time	Educational attainment
B&NS	13204	39687	39531	39422
B&S	5311	16093	16021	15990
NB&NS	4362	14015	13937	13919
NB&S	2568	8488	8430	8419
Sum	25445	78283	77919	77750

B&NS: breastfed and not exposed to maternal smoking around birth,

B&S: breastfed and exposed to maternal smoking around birth,

NB&NS: not breastfed and not exposed to maternal smoking around birth, and

NB&S: not breastfed and exposed to maternal smoking around birth.

In a preliminary analysis, we estimated the proportion of the phenotypic variance and the genetic correlation explained by genome-wide SNPs for fluid intelligence, memory, reaction time and educational attainment (Table 2). We used a four-trait GREML^21^ that explicitly modelled the genetic as well as residual covariance structure, as there were multiple phenotypes for each individual (see Methods). Adjusted phenotypes controlled for non-genetic confounders were used (see Methods). All of the estimates were significantly different from zero, indicating that there were significant genetic factors underlying those traits. The estimated SNP-heritability ranged from 0.067 to 0.219, and the estimated genetic correlations ranged from −0.338 to 0.674. The estimates agreed approximately with those in Davies *et al*.^26^ although we used a different subsets of the sample of phenotypes, different SNPs, and a different model for analysis (see Methods).

**Table 2:** The proportion of the phenotypic variance (diagonal) and genetic correlation (upper diagonal) explained by genome-wide SNPs and phenotypic correlation (lower diagonal).

	Fluid intelligence	Memory	Reaction time	Educational attainment
Fluid intelligence (N = 25445)	0.219 (0.013)	−0.338 (0.043)	−0.198 (0.042)	0.674 (0.027)
Memory (N = 78283)	−0.125 (0.006)	0.067 (0.004)	0.129 (0.044)	−0.320 (0.032)
Reaction time (N = 77919)	−0.118 (0.006)	0.064 (0.003)	0.080 (0.005)	−0.084 (0.031)
Educational attainment (N = 77750)	0.395 (0.005)	−0.115 (0.003)	−0.083 (0.003)	0.177 (0.005)

**Figure 1:**
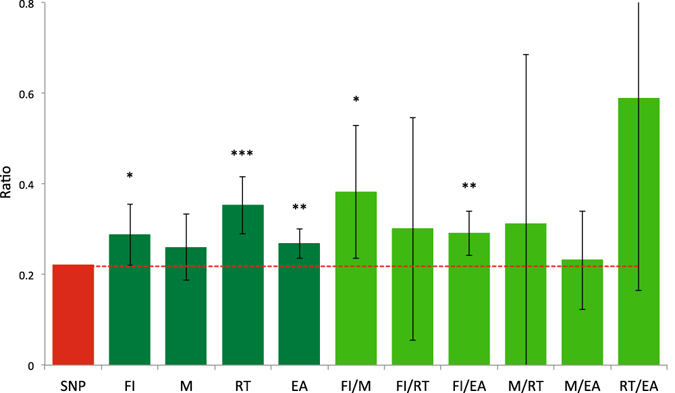
The ratio of the genetic variance and covariance explained by CNS SNPs over the total genetic variance and covariance from the annotation analyses. Vertical error bar is 95% confidence interval. The ratio of SNPs attributed to genes in the CNS is also shown (red). FI: Fluid intelligence. M: Memory RT: Reaction time EA: Educational attainment.

We further partitioned the genetic variance and covariance into three functional categories; SNPs in genes that were differentially expressed in the central nervous system (CNS); SNPs in the other genes; and the remaining SNPs (see Methods). Figure 1 shows that the proportion of the genetic variance for fluid intelligence explained by the CNS SNPs was significantly higher than expected for the same number of random SNPs (ratio = 0.289, p-value = 3.3E-02), reaction time (0.352, 3.4E-05) and educational attainment (0.269, 2.7E-03). Figure 1 also shows that the proportion of the genetic covariance between fluid intelligence and memory explained by the CNS SNPs was significantly greater than expected based on SNP number (ratio = 0.382, p-value = 2.8E-02), and similarly for the genetic covariance between fluid intelligence and educational attainment (0.291, 4.0E-03).

**Figure 2:**
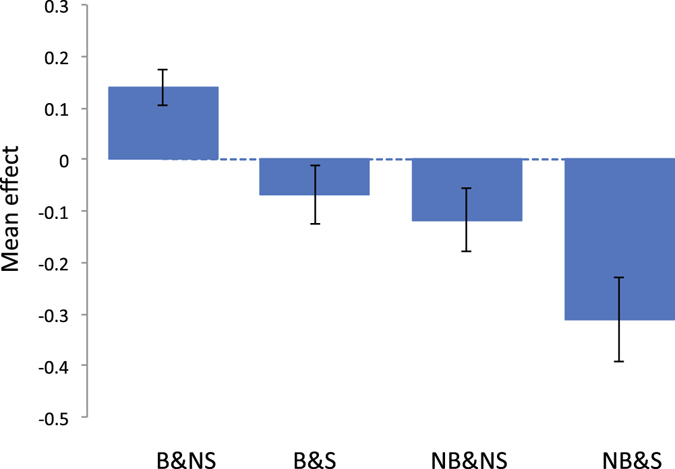
Mean trait value of fluid intelligence for each group classified by breastfeeding and maternal smoking status. Vertical bar is 95% confidence interval. The phenotypes of fluid intelligence were adjusted for birth year, age at recruitment, sex, assessment centre, genotype measurement batch and 15 principal components.

**Figure 3:**
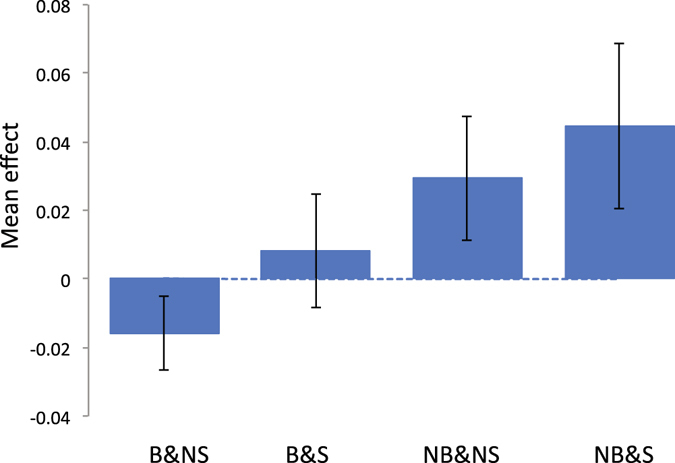
Mean trait value of memory (number of incorrect matches) for each group classified by breastfeeding and maternal smoking status. Vertical bar is 95% confidence interval. The phenotypes of memory were adjusted for birth year, age at recruitment, sex, assessment centre, genotype measurement batch and 15 principal components.

**Figure 4:**
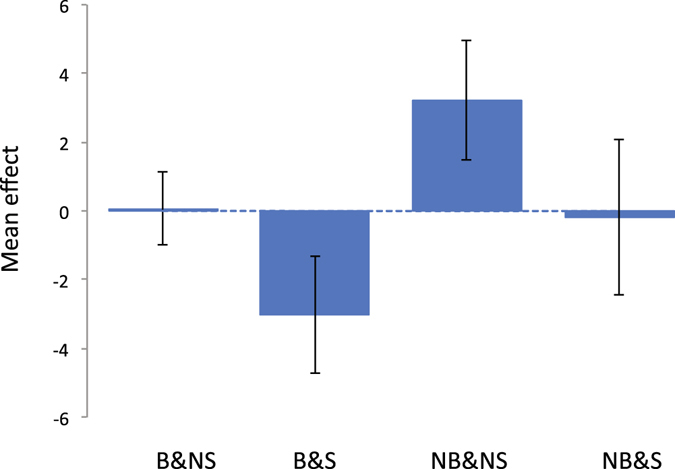
Mean trait value of reaction time for each group classified by breastfeeding and maternal smoking status. Vertical bar is 95% confidence interval. The phenotypes of reaction time were adjusted for birth year, age at recruitment, sex, assessment centre, genotype measurement batch and 15 principal components.

**Figure 5:**
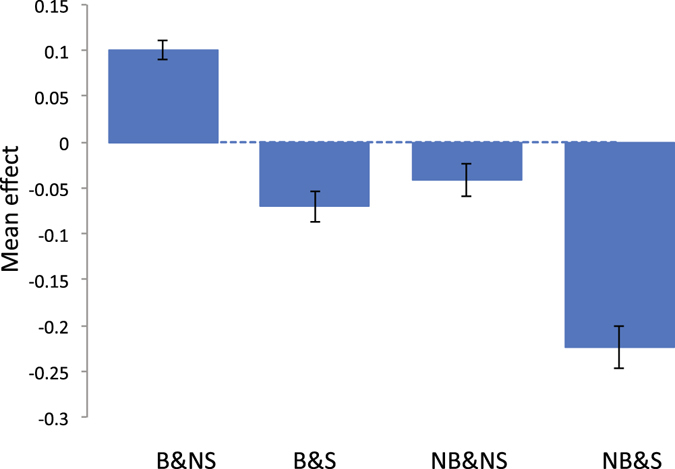
Mean trait value of educational attainment for each group classified by breastfeeding and maternal smoking status. Vertical bar is 95% confidence interval. The phenotypes of educational attainment were adjusted for birth year, age at recruitment, sex, assessment centre, genotype measurement batch and 15 principal components.

Next, we stratified the individuals according to the status of breastfeeding and maternal smoking around birth, i.e. breastfeeding and non-smoking (B&NS), breastfeeding and smoking (B&S), non-breastfeeding and non-smoking (NB&NS), non-breastfeeding and smoking (NB&S) as in Table 1. Trait means and range (95% CI) are shown in Figs 2–5. The patterns show clearly that the cognitive function or educational performance is increased when individuals were breastfed, and decreased when there was maternal smoking around birth for all traits except reaction time.

To estimate genome-wide G × E we used a four-variate GREML to estimate genetic variance and covariance between the four environmental groups, i.e. B&NS, B&S, NB&NS and NB&S. We found that there was significant G × E between B&NS and NB&NS for both fluid intelligence (p-value = 1.0E-03) and educational attainment (p-value = 8.3E-05) (Tables 3 and 4). The genetic correlation between two environments (B&NS and NB&NS) was 0.597 (SE 0.123) and 0.748 (SE 0.064) for fluid intelligence and educational attainment, respectively (Tables 3 and 4). There was also significant G × E between B&S and NB&NS for fluid intelligence (p-value = 3.9E-05) with a genetic correlation of 0.345 (SE 0.159) (Table 3). Even after correcting for multiple testing (4 traits each with 6 contrasts between environments where the corrected threshold is p = 0.05/24 = 0.002), these interactions remained significant (Tables 3 and 4). We also used permutation tests and confirmed the level of significance (Supplementary Figures 1, 2 and 3). There was no evidence of G × E for memory and reaction time (Supplementary Tables 1 and 2), which was probably due to the fact that the genetic variance was low (Table 2) such that the power to detect G × E is reduced.

**Table 3:** The proportion of the phenotypic variance and genetic correlation between the status of breastfeeding and maternal smoking around birth for fluid intelligence.

	Estimate	SE	P-value	
*h* ^*2*^ for B&NS	0.219	0.025	6.5E-19^a^	***
*h* ^*2*^ for B&S	0.260	0.059	9.9E-06^a^	***
*h* ^*2*^ for NB&NS	0.366	0.073	5.9E-07^a^	***
*h* ^*2*^ for NB&S	0.139	0.117	2.3E-01^a^	
*r*_*G*_ (B&S, B&NS)	0.931	0.149	6.4E-01^b^	
*r*_*G*_ (NB&NS, B&NS)	0.597	0.123	1.0E-03^b^	**
*r*_*G*_ (NB&NS, B&S)	0.345	0.159	3.9E-05^b^	***
*r*_*G*_ (NB&S, B&NS)	1.213	0.546	7.0E-01^b^	
*r*_*G*_ (NB&S, B&S)	1.202	0.577	7.3E-01^b^	
*r*_*G*_ (NB&S, NB&NS)	1.060	0.526	9.1E-01^b^	

^a^Testing if the estimate is different from 0.

^b^Testing if the estimate is different from 1; The genetic correlations (*r*_*G*_) between NB&NS and B&NS, and NB&NS and B&S are significantly different from 1 as an evidence of G × E. Even after a multiple testing correction (p-value threshold = 0.05/24 = 0.002), these interactions remained significant.

^***^P-value < 0.001; ^**^P-value < 0.01; ^*^P-value < 0.05.

**Table 4:** The proportion of the phenotypic variance and genetic correlation between the status of breastfeeding and maternal smoking around birth for educational attainment.

	Estimate	SE	P-value	
*h* ^*2*^ for B&NS	0.184	0.009	4.0E-87^a^	***
*h* ^*2*^ for B&S	0.169	0.020	4.2E-17^a^	***
*h* ^*2*^ for NB&NS	0.212	0.023	3.9E-20^a^	***
*h* ^*2*^ for NB&S	0.163	0.037	8.4E-06^a^	***
*r*_*G*_ (B&S, B&NS)	0.912	0.073	2.3E-01^b^	
*r*_*G*_ (NB&NS, B&NS)	0.748	0.064	8.3E-05^b^	***
*r*_*G*_ (NB&NS, B&S)	0.866	0.099	1.8E-01^b^	
*r*_*G*_ (NB&S, B&NS)	1.013	0.129	9.2E-01^b^	
*r*_*G*_ (NB&S, B&S)	1.090	0.167	5.9E-01^b^	
*r*_*G*_ (NB&S, NB&NS)	0.929	0.153	6.4E-01^b^	

^a^Testing if the estimate is different from 0.

^b^Testing if the estimate is different from 1; The genetic correlation (*r*_*G*_) between NB&NS and B&NS is significantly different from 1 as an evidence of G × E. Even after a multiple testing correction (p-value threshold = 0.05/24 = 0.002), the interaction remained significant.

^***^P-value < 0.001; ^**^P-value < 0.01; ^*^P-value < 0.05.

For fluid intelligence and educational attainment that showed a significant G × E signal, we further estimated genome-wide G × E in a sex-stratified analyses to see if there was any significant sex difference in each environment, i.e. to test if genetic correlation between males and females was significantly different from 1. We found no significant difference between males and females in the same environment for all cases except that there was a weak signal of difference between male and female for NB&S group for fluid intelligence (p-value = 3.3E-02) (Supplementary Tables 3 and 4). After correcting for multiple testing, there was no evidence for sex difference (Supplementary Tables 3 and 4).

We compared genome-wide association studies (GWAS) based on the different environmental groups for fluid intelligence or educational attainment between which there was significant G × E. In the GWAS based on B&NS, there were no genome-wide significant SNP whereas GWAS based on NB&NS detected a significant locus in chromosome 22 for fluid intelligence (Supplementary Figure 4). For educational attainment, the GWAS based on B&NS detected no genome-wide significant SNP on chromosome 17 whereas significant SNPs were found in data from the NB&NS (Supplementary Figure 5).

Finally, we assessed the effect of genome-wide G × E in the accuracy of genomic prediction, i.e. precision medicine^27^. We randomly selected 1000 target samples within a particular environmental group, and predicted their phenotypes using genome-wide SNP effects estimated in two different discovery data sets, one sampled from the same environmental group and the other sampled from a different environmental group. The two discovery sets had the same sample size, and there was no overlap between the target and any of the discovery data sets. The analyses between B&NS and NB&NS for fluid intelligence (Supplementary Figure 6) or educational attainment (Supplementary Figure 7) and the analysis between B&S and NB&NS for fluid intelligence (Supplementary Figure 8) showed that the prediction accuracies were significantly higher when using the target and discovery sample from the same environmental group than when using the target and discovery sample from different environmental groups. These results supported our finding of genome-wide G × E, and has an important implication for genomic prediction strategies.

## Discussion

We reported significant genome-wide G × E of fluid intelligence and educational attainment conditional on breastfeeding and maternal smoking status. To our knowledge, this is the first study to explore whether genetic effects of later cognitive performance interact with maternal environments using independent unrelated samples and based on genomic data. There have been a number of studies that investigated the environmental effect of breastfeeding or maternal smoking status on cognitive performance^1–3, 5, 10–12^, which was, however, limited to find out simple additive effects and their difference between environments. In this study, we investigated G × E of later cognitive performance and educational attainment using a novel design and a statistical approach based on genomic data that enables to link the same genotypes across different environments.

The analyses of the four traits (Table 2) or those of the four environmental groups within each trait (Tables 3 and 4) could be done efficiently in a four-trait or four-variate GREML analysis using MTG2 software^21^. The four-variate linear mixed model, which can fit four response variables simultaneously, is computationally faster and has higher accuracy and power, compared to a number of separate analyses of bi-variate models. For the analyses of the four traits (Table 2), we explicitly modelled a residual covariance structure as each individual has multiple phenotypes measured for the four traits. In the four-trait analyses, we were interested in testing whether the different traits had shared genetic effects (i.e. testing if *r*_*G*_ is significantly different from 0). For the analyses of G × E for each trait, we tested if the different environment groups were heterogeneous (i.e. testing if *r*_*G*_ is significantly different from 1, hence G × E). In our G × E analyses, there was no need to model residual covariance structure because it was not possible for the same individual to have multiple measures across the environments.

For fluid intelligence and educational attainment, although we found evidence for G × E between B&NS and NB&NS, and B&S and NB&NS, there was no signal for that between B&NS and NB & S, even though these environments showed the highest contrast in mean effects (Figs 2–5). This was probably due to the fact that the samples size for NB & S was low (Table 1) therefore there might be less power for the group. Indeed, the SNP-heritability of fluid intelligence for the NB & S group was not significantly different from 0.

We carried out the analyses on the four environments classified by the combined effects of breastfeeding and maternal smoking status (Tables 3 and 4). We also explored G × E analyses with each exposure of the main effects separately (Supplementary Tables 5 and 6). There was a significant evidence of G × E for fluid intelligence and educational attainment when comparing the group with and without breastfeeding (p-value = 0.0048 and 0.042 in Supplementary Table 5) although there was no evidence when using maternal smoking status. In those analyses considering each exposure separately (breastfeeding or maternal smoking), there was a possibility of confounded environmental factors (i.e. the effects of maternal smoking and breastfeeding were partially confounded) could dilute G × E effects (compare Table 3 and Supplementary Table 5). For this reason it is important to assess G × E on the four unique environmental conditions. We should also emphasise that the availability of genomic data allowed a much more flexible statistical approach for investigating G × E across various environmental groupings.

To ensure that socio-economic status were not affecting our results, we also undertook the analyses with phenotypes additionally adjusted for average total household income. Supplementary Tables 7–9 shows that the signals for G x E were still significant even though a less significant signal was expected partly because the sample size was reduced due to missing information on income (Supplementary Table 7). This indicates that the effects of breastfeeding and maternal smoking cannot be fully explained by socio economic status.

We showed that the accuracy of genomic prediction, which is an emerging tool in the personalised or precision medicine^27^, could be significantly decreased if there was G × E and the discovery and target samples had different environmental conditions (Supplementary Figures 6–8). To increase the accuracy, environment conditions for sample should be carefully considered, i.e. recorded where possible and considered in the statistical analysis. The same holds for GWAS where the effects of causal genetic variants could be environment-specific (Supplementary Figures 4 and 5).

Except for one trait (memory), all of the cognitive phenotypes in the UK Biobank data have been reported to have a reasonable reliability from re-test data^28^. Intraclass correlation between the first and second wave of re-test data was 0.65, 0.16 and 0.57 for fluid intelligence, memory and reaction time, respectively^28^. In order to check the quality of environmental measures, we obtained a coefficient of determination (R^2^) using a regression with the sex or year-of-birth information as a dependent variable and the ‘known’ and ‘unknown’ answer as an explanatory variable. Supplementary Table 10 shows that little variance is explained by the sex or age difference for the ‘unknown’ answer. In fact, for all the analyses in our study, the effects of sex, year-of-birth and age-at-recruitment were appropriately adjusted in the models. Moreover, we explicitly checked our results with sex stratified analyses and found negligible sex difference (Supplementary Tables 3 and 4). Furthermore, we checked whether the breastfeeding responses by age are consistent with broader UK trends at the time (and the same for maternal smoking, i.e. rates of female smoking). The breastfeeding rate was decreased over the 1950–60 period and it was 51% in 1975 in UK^29, 30^, which approximately conform with the pattern of the rate from the UK Biobank data (Supplementary Table 11). Female smoking rate was ~40%^31^ between 1940 and 1970, which was not too dissimilar to the maternal smoking rate in the UK Biobank data (Supplementary Table 11). It should be noted that maternal smoking would be lower than average female smoking rate.

We showed that for fluid intelligence, memory and educational attainment, the mean score of the individuals who were breastfed and not exposed to maternal smoking around birth was significantly higher than those who were not breastfed and exposed to maternal smoking around birth (Figs 2–5), which agrees with previous studies^1–3, 6–9^. A limitation of our study was that we did not explicitly adjust for maternal intelligence due to lack of information. A number of studies reported that significant effects of breastfeeding or maternal smoking disappeared after adjusting maternal intelligence^4, 5, 10, 11^. However, it is also possible that a stringent adjustment of confounding effects such as maternal intelligence can over-correct the true effects of breastfeeding or maternal smoking. Indeed, breastfeeding effects on cognitive function or brain development have been evidenced in experimental species^32^ and brain image analyses^33, 34^ using experiments without such confounding. Some studies reported significant breastfeeding effects even after correcting for confounding^35, 36^. Maternal smoking effects on cognitive function or brain development have also been demonstrated in experimental species^37^ and brain image analyses^38^ that were without confounding effects. Another limitation was that we did not consider gestational age in the analyses because the information was not available. There are a number of studies reporting gestational age is a risk factor for later cognitive development^39, 40^. A further study is required to confirm the combined effects of breastfeeding and maternal smoking around birth after an appropriate adjustment of confounding effects such as maternal intelligence and gestational age. Nevertheless, our primary aim was to estimate G × E based on the four groups stratified according to breastfeeding and maternal smoking status in the variance component approach. Even when the dependent variable within each of the four stratified groups is standardised, i.e. a mean of zero and a variance of one, which can correct the mean difference for confounding effects, it would not affect the resulting estimated genetic correlation between environments.

In summary, breastfeeding and maternal smoking status are important environmental factors in fluid intelligence, memory and educational attainment. More importantly, we showed that the genetic expression of fluid intelligence and educational attainment differs for different maternal environments, implying that these environments interact with gene action. In the presence of G × E, genome-wide significant SNPs were different between different environmental groups. Genomic prediction accuracies were significantly higher when using the target and discovery sample from the same environmental group than when using those from different environmental groups. This finding has important implications in future studies of the genetic architecture, genomic prediction and GWAS for later cognitive performances.

## Methods

### Data

We used the UK Biobank database (http://www.ukbiobank.ac.uk)^25^. UK Biobank Research Ethics Committee (REC) approval number is 11/NW/0382. Our reference number approved by UK Biobank is 14575 and we confirm that all experiments were performed in accordance with relevant guidelines and regulations. There are 502,648 participants, who were aged from 40–69 years and recruited between 2006 and 2010, measured for various complex traits and variables including cognitive functions and environmental factors. We used three cognitive traits, fluid intelligence, memory ability and reaction time, and educational attainment for which the participants were grouped into four classes according to the status of breastfeeding and maternal smoking around birth, i.e. breastfed and non-smoking (B&NS), breastfed and smoking (B&S), non-breastfed and non-smoking (NB&NS) and non-breastfed and smoking (NB&S).

### Fluid intelligence

This measure included data on questions designed to assess the cognitive function to solve problems that require logic and reasoning ability (http://biobank.ctsu.ox.ac.uk/crystal/field.cgi?id=20016). The participant had a limited time (2 minutes) to complete as many questions as possible from the test presented in a touchscreen computer. There were 184,487 participants measured for the trait.

### Memory

Memory test included data on ‘pairs’ matching task on a touchscreen computer (http://biobank.ctsu.ox.ac.uk/crystal/field.cgi?id=399). Participants were asked to remember the location of six matching pairs of cards. Then, the cards were then turned face down on the screen and the participant had to select correct cards that match the original pairs in the fewest tries. Number of incorrect matches was recorded. There were 498,545 participants measured for the trait.

### Reaction time

Reaction time test involved data on a test to assess reaction time and was based on 12 rounds of the card-game ‘Snap’ (http://biobank.ctsu.ox.ac.uk/crystal/field.cgi?id=20023). The computer screen showed two cards at a time; if both cards were the same, the participants were asked to press a button-box that was on the table in front of them as quickly as possible. This reaction time was recorded. There were 496,902 participants measured for the trait.

### Educational attainment

The educational categories were divided into 7 groups: College or University degree; A-levels/AS-levels; O-levels; CSEs or equivalent; NVQ or HND or HNC or equivalent; other professional qualifications, e.g. nursing, teaching; none (http://biobank.ctsu.ox.ac.uk/crystal/field.cgi?id=6138). Following Guggenheim *et al*.^41^, we reduced to 4 categories to consider approximately equal years of academic education, that is (1) None; (2) O-levels or CSEs; (3) A-levels, NVQ, HND, HNC or other professional qualification; (4) College or University degree. There were 498,528 participants measured for the trait.

### Breastfed as a baby and maternal smoking around birth

As early life environmental factors, self-reported breastfed and maternal smoking status were available. Breastfeeding status was available for 501,748 participants (294,708 for yes, 111,381 for no, 125,114 for unknown and 471 for no answer). By touch screen interview, breastfeeding status was recorded in response to the question “Were you breastfed when you were a baby?”. There was no detailed measure for the amount of breast-milk intake.

Maternal smoking status around birth was available for 494,400 participants (134,141 for yes, 324,894 for no, 64,596 for unknown and 255 for no answer). Through a screen touch interview, maternal smoking status was recorded in response to the question “Did your mother smoke regularly around the time when you were born?”. There was no detailed measure for the amount of smoking.

### Genotypic information

For 502,648 participants, 152,249 individuals were genotyped and available for ~70 millions SNPs after a standard imputation process (for more details, see http://biobank.ctsu.ox.ac.uk/crystal/refer.cgi?id=157020). We further applied stringent quality control (QC) with an imputation r-squared > 0.6, MAF > 0.01, H-W test p-value < 0.0001, SNP missingness > 0.05 and individual missingness > 0.05. Furthermore, we selected high quality HapMap3 SNPs that were reliable in estimating genetic variance and covariance at the genome-wide level, feasible for more complicated analyses and there was no substantial difference between estimated genetic variances from HapMap3 and 1000 genome SNPs^17, 42–44^. After QC, 931,295 HapMap3 SNPs were remained for the analyses.

### Individual QC

To make sure there was no biased estimate in genetic covariance due to confounders, we excluded non-British ancestry within self-identified British according to genetic principal components, and used individuals defined as Caucasian in the genetic ethnic grouping data. We further excluded high relatedness (pair-wise relationship > 0.05). After QC and matching breastfeeding and maternal smoking status, a sample of 25,445, 78,283, 77,919 and 77,750 genotyped and phenotyped individuals was remained for analysing fluid intelligence, memory, reaction time and educational attainment (Table 1).

All phenotypes were adjusted for birth year, age at recruitment, sex, assessment centre, genotype measurement batch and 15 principal components to control for confounding non-genetic effects before all analyses using a linear regression. Inverse normal transformation was further applied to the adjusted phenotypes for memory, reaction time and educational attainment of which distribution was skewed or non-normal (Supplementary Table 12) to satisfy a normality assumption for the multi-variate GREML analyses.

### G×E model using multi-variate GREML for each trait

We propose a statistical approach that integrates a novel design of unrelated subjects across different environmental conditions in which repeated measures of the same individual are typically unavailable (such as breastfeeding and maternal smoking status). In the approach, we estimate genetic variance and covariance for a trait of interest across multiple environmental conditions that are tagged by SNPs to provide estimate of genome-wide G × E, i.e. in presence of G × E, the genetic correlation between traits measured in different environments is significantly lower than one^23^. Covariance structure between unrelated subjects can be constructed based on genome-wide SNPs^18, 45^. The model can be written as

$$\begin{array}{c}{{\bf{y}}}_{1}={{\bf{X}}}_{1}{{\bf{b}}}_{1}+{{\bf{Z}}}_{1}{{\bf{g}}}_{1}+{{\bf{e}}}_{1}\quad \,\,\,{\rm{for}}\,{\rm{B}}{\&}{\rm{NS}}\,{\rm{group}}\\ {{\bf{y}}}_{2}={{\bf{X}}}_{2}{{\bf{b}}}_{2}+{{\bf{Z}}}_{2}{{\bf{g}}}_{2}+{{\bf{e}}}_{2}\quad \,{\rm{for}}\,{\rm{B}}{\&}{\rm{S}}\,{\rm{group}}\\ {{\bf{y}}}_{3}={{\bf{X}}}_{3}{{\bf{b}}}_{3}+{{\bf{Z}}}_{3}{{\bf{g}}}_{3}+{{\bf{e}}}_{3}\quad \,\,{\rm{for}}\,{\rm{NB}}{\&}{\rm{NS}}\,{\rm{group}}\\ {{\bf{y}}}_{4}={{\bf{X}}}_{4}{{\bf{b}}}_{4}+{{\bf{Z}}}_{4}{{\bf{g}}}_{4}+{{\bf{e}}}_{4}\quad \,\,{\rm{for}}\,{\rm{NB}}{\&}{\rm{S}}\,{\rm{group}}\end{array}$$

where **y** are four column vectors of phenotypic observation, each vector belongs to each environmental condition, **b** are four vectors of fixed effects, **g** are four vectors of additive genetic effects and **e** are four vectors of residuals. The random effects (**g** and **e**) are assumed to be normally distributed with mean zero. **X** and **Z** are incidence matrices for the effects **b** and **g**, respectively. The variance covariance matrix is defined as

$${\bf{V}}=[\begin{array}{ccc}{\bf{Z}}{\bf{A}}{\sigma }_{{g}_{1}}^{2}{\bf{Z}}\text{'}+{\bf{I}}{\sigma }_{{e}_{1}}^{2} & \ldots  & {\bf{Z}}{\bf{A}}{\sigma }_{{g}_{1,4}}{\bf{Z}}\text{'}\\ \vdots  & \ddots  & \vdots \\ {\bf{Z}}{\bf{A}}{\sigma }_{{g}_{1,4}}{\bf{Z}}\text{'} & \cdots  & {\bf{Z}}{\bf{A}}{\sigma }_{{g}_{4}}^{2}{\bf{Z}}\text{'}+{\bf{I}}{\sigma }_{{e}_{4}}^{2}\end{array}]$$

where **A** is the genomic similarity matrix based on genome-wide SNPs^18, 45^, and **I** is an identity matrix. The terms, ${\sigma }_{{g}_{i}}^{2}$ and ${\sigma }_{{e}_{i}}^{2}$ denote the genetic and residual variance in the *i*th environment, and ${\sigma }_{{g}_{i,j}}$ the genetic covariance of the environment *i* and *j*. It is assumed that there is no residual covariance because individual has no repeated measures. The variance and covariance components were estimated by a multi-trait or multivariate GREML. The genetic correlation was the ratio of the covariance scaled by the square root of the product of the variances between two environmental groups. The variance of the ratio was obtained by the Delta method using the information matrix^24^, and used to assess the significance of the estimate being different from 1, an evidence of G × E.

### Unbiased estimation of the genetic correlation between two groups with truncated selections

Assuming that a random variable *y* is distributed as *N* (0, 1), a linear model can be written as

$$y=g+e$$

where *g* is random genetic effects, which are distributed as *N* (0, *h* ^*2*^), and *e* is random residuals, which are from *N* (0, 1-*h* ^*2*^). When the phenotype *y* is selected such that values less than a threshold *t*_*1*_ and more than another threshold *t*_*2*_ are selected. Then, the variables after the selection can be written as

$${y}_{s}={g}_{s}+{e}_{s}$$

Following quantitative genetic theory^23^, the mean and variance for the selected variable are

$$E({y}_{s})=({K}_{2}{i}_{2}-{K}_{1}{i}_{1})/({K}_{2}-{K}_{1}),$$

$$\begin{array}{c}E({y}_{s}^{2})=[{K}_{2}(1+{i}_{2}{t}_{2})-{K}_{1}(1+{i}_{1}{t}_{1})]/({K}_{2}-{K}_{1})\,{\rm{and}}\\ var({y}_{s})=E({y}_{s}^{2})-E{({y}_{s})}^{2}=\frac{({K}_{2}-{K}_{1})[{K}_{2}(1+{i}_{2}{t}_{2})-{K}_{1}(1+{i}_{1}{t}_{1})]-{({K}_{2}{i}_{2}-{K}_{1}{i}_{1})}^{2}}{{({K}_{2}-{K}_{1})}^{2}}\end{array}$$

The mean and variance for the genetic values after the selection are

$$E({g}_{s})=E({y}_{s}){h}^{2},$$

1 $$\begin{array}{c}E({g}_{s}^{2})={h}^{2}[{K}_{2}(1+{h}^{2}{i}_{2}{t}_{2})-{K}_{1}(1+{h}^{2}{i}_{1}{t}_{1})]/({K}_{2}-{K}_{1})\,{\rm{and}}\\ var({g}_{s})=E({g}_{s}^{2})-E{({g}_{s})}^{2}=\frac{{h}^{2}[({K}_{2}-{K}_{1})[{K}_{2}(1+{h}^{2}{i}_{2}{t}_{2})-{K}_{1}(1+{h}^{2}{i}_{1}{t}_{1})]-{h}^{2}{({K}_{2}{i}_{2}-{K}_{1}{i}_{1})}^{2}]}{{({K}_{2}-{K}_{1})}^{2}}\end{array}$$

The heritability after the selection is

$${h}_{s}^{2}=\frac{{h}^{2}[({K}_{2}-{K}_{1})[{K}_{2}(1+{h}^{2}{i}_{2}{t}_{2})-{K}_{1}(1+{h}^{2}{i}_{1}{t}_{1})]-{h}^{2}{({K}_{2}{i}_{2}-{K}_{1}{i}_{1})}^{2}]}{({K}_{2}-{K}_{1})[{K}_{2}(1+{i}_{2}{t}_{2})-{K}_{1}(1+{i}_{1}{t}_{1})]-{({K}_{2}{i}_{2}-{K}_{1}{i}_{1})}^{2}}.$$

From Eq. (1), the genetic values after the selection can be defined as^20, 46^

2 $${g}_{s}=c+bg$$

where *c* is a constant and $b=\sqrt{\frac{({K}_{2}-{K}_{1})[{K}_{2}(1+{h}^{2}{i}_{2}{t}_{2})-{K}_{1}(1+{h}^{2}{i}_{1}{t}_{1})]-{h}^{2}{({K}_{2}{i}_{2}-{K}_{1}{i}_{1})}^{2}}{{({K}_{2}-{K}_{1})}^{2}}}$.

From Eq. (2), the genetic covariance between two sets of selected sample can be written as

$${\rm{cov}}({g}_{s1},{g}_{s2})={b}_{1}{b}_{2}{\rm{cov}}({g}_{1},{g}_{2}),$$

and the genetic correlation is

3 $${\rm{cor}}({g}_{s1},{g}_{s2})=\frac{{b}_{1}{b}_{2}{\rm{cov}}({g}_{1},{g}_{2})}{{b}_{1}{\rm{var}}({g}_{1}){b}_{2}{\rm{var}}({g}_{2})}={\rm{cor}}({g}_{1},{g}_{2})$$

Therefore, from equation (3), it is clear that even when samples are ascertained with a truncated selection, the genetic correlation is unbiased, and there is no spurious estimation of G × E.

For estimation of genetic correlation between two groups classified by an environmental variable (*y*_*E*_) that is correlated with a trait of interest, the mean and variance for the selected variable for the trait are

$$E({y}_{s})=r({K}_{2}{i}_{2}-{K}_{1}{i}_{1})/({K}_{2}-{K}_{1}),$$

where *r* is phenotypic correlation between the trait and environmental variable,

$$\begin{array}{c}E({y}_{s}^{2})=[{K}_{2}(1+{r}^{2}{i}_{2}{t}_{2})-{K}_{1}(1+{r}^{2}{i}_{1}{t}_{1})]/({K}_{2}-{K}_{1})\,{\rm{and}}\\ \begin{array}{l}var({y}_{s})=E({y}_{s}^{2})-E{({y}_{s})}^{2}=\frac{({K}_{2}-{K}_{1})[{K}_{2}(1+{r}^{2}{i}_{2}{t}_{2})-{K}_{1}(1+{r}^{2}{i}_{1}{t}_{1})]-{r}^{2}{({K}_{2}{i}_{2}-{K}_{1}{i}_{1})}^{2}}{{({K}_{2}-{K}_{1})}^{2}}\end{array}\end{array}$$

The mean and variance for the genetic values after the selection based on correlated environmental variable are

$$E({g}_{s})=\frac{{\rm{cov}}(g,{g}_{E})}{{\rm{cov}}(y,{y}_{E})}E({y}_{s})=0,$$

where *g*_*E*_ is genetic effects for the environmental variable, which should be zero,

4 $$\begin{array}{c}E({g}_{s}^{2})={h}^{2}[{K}_{2}(1+{h}^{2}{r}^{2}{i}_{2}{t}_{2})-{K}_{1}(1+{h}^{2}{r}^{2}{i}_{1}{t}_{1})]/({K}_{2}-{K}_{1})\,{\rm{and}}\\ {\rm{var}}({g}_{s})=E({g}_{s}^{2})-E{({g}_{s})}^{2}=\frac{{h}^{2}[({K}_{2}-{K}_{1})\{{K}_{2}(1+{h}^{2}{r}^{2}{i}_{2}{t}_{2})-{K}_{1}(1+{h}^{2}{r}^{2}{i}_{1}{t}_{1})\}]}{{({K}_{2}-{K}_{1})}^{2}}\end{array}$$

The heritability after the selection is

$${h}_{s}^{2}=\frac{{h}^{2}[({K}_{2}-{K}_{1})\{{K}_{2}(1+{h}^{2}{r}^{2}{i}_{2}{t}_{2})-{K}_{1}(1+{h}^{2}{r}^{2}{i}_{1}{t}_{1})\}]}{({K}_{2}-{K}_{1})[{K}_{2}(1+{r}^{2}{i}_{2}{t}_{2})-{K}_{1}(1+{r}^{2}{i}_{1}{t}_{1})]-{r}^{2}{({K}_{2}{i}_{2}-{K}_{1}{i}_{1})}^{2}}$$

From Eq. (4), the genetic values after the selection based on correlated environmental variable can be defined as^20, 46^

5 $${g}_{s}=c+bg$$

where *c* is a constant and $b=\sqrt{\frac{[({K}_{2}-{K}_{1})\{{K}_{2}(1+{h}^{2}{r}^{2}{i}_{2}{t}_{2})-{K}_{1}(1+{h}^{2}{r}^{2}{i}_{1}{t}_{1})\}]}{{({K}_{2}-{K}_{1})}^{2}}}$.

Therefore, as the same as in Eq. (3), it is clear that the genetic correlation is unbiased because of the selection based on correlated environmental variable. We also confirm this with a simulation (result not shown).

### Permutation p-value

We obtained a p-value using the Wald test; assuming that the distribution of estimated genetic correlation was normal. We also carried out permutation tests to infer an empirical distribution of estimated genetic correlation, and estimated permutation p-value for the case of significant G × E found by the Wald test. In the permutation test, the environmental status was randomly shuffled, and genetic correlation between two environments was estimated. It was noted that the number of phenotypic records for the first and second environment in the permutation test was kept as the same as in the original data structure. The number of permutation tests was 1000 for each case.

### Sex-stratified analyses

For the case of significant G × E, it was of interest to see if there was any significant sex interaction. We used eight-variate GREML for each trait that could fit eight groups, i.e. B&NS_M_, B&NS_F_, B&S_M_, B&S_F_, NB&NS_M_, NB&NS_F_, NB&S_M_ and NB&NS_F_.

### Genomic prediction

For the case of significant G × E, we carried out predicting phenotypes in a subset of sample from an environment group using the genome-wide SNP effects estimated in another subset of sample either from the same or from different environmental group. For the target sample (to be predicted for their phenotypes), we randomly selected 1000 individuals for both fluid intelligence and educational attainment. For the discovery sample (to estimate the genome-wide SNP effects), we used 3362 individuals for fluid intelligence and 12919 for educational attainment that were randomly selected either from the same environmental group as the target sample or from a different environmental group. In estimating the genome-wide SNP effects, we used GBLUP^21^. There was no overlapped sample between the target and discovery sample. The prediction accuracy was obtained from the correlation between predicted and true phenotypes in the target data.

### Genome-wide association study

We compared genome-wide significant SNPs based on different environmental groups between which there was significant G × E. We used plink software^47^ to obtain GWAS p-values that were plotted using qqman software^48^.

### Estimation of genetic variance and covariance between the four traits

While our primary interest was to estimate G × E, it was of interest to dissect shared genetic architecture between the cognitive traits as preliminary analyses. We used a multi-trait GREML that fitted four traits simultaneously to estimate the genetic and residual variances and covariance between the four traits. It is noted that because individuals had multiple phenotypes, residual covariance was explicitly modelled, otherwise the genetic covariance would be inflated. The details of model can be found elsewhere^21, 22^. Briefly, the model is very similar to the G × E model above except that **y** are four column vectors of phenotypic observation, each vector belongs to each trait, and the variance covariance matrix has an additional term of residual covariance as,

$${\bf{V}}=[\begin{array}{ccc}{\bf{Z}}{\bf{A}}{\sigma }_{{g}_{1}}^{2}{\bf{Z}}\text{'}+{\bf{I}}{\sigma }_{{e}_{1}}^{2} & \ldots  & {\bf{Z}}{\bf{A}}{\sigma }_{{g}_{1,4}}{\bf{Z}}\text{'}+{\bf{I}}{\sigma }_{{e}_{1,4}}\\ \vdots  & \ddots  & \vdots \\ {\bf{Z}}{\bf{A}}{\sigma }_{{g}_{1,4}}{\bf{Z}}\text{'}+{\bf{I}}{\sigma }_{{e}_{1,4}} & \cdots  & {\bf{Z}}{\bf{A}}{\sigma }_{{g}_{4}}^{2}{\bf{Z}}\text{'}+{\bf{I}}{\sigma }_{{e}_{4}}^{2}\end{array}]$$

where the other terms are defined as above and ${\sigma }_{{e}_{i,j}}$ the residual covariance of the trait *i* and *j*.

We were also interested in finding if the central nervous system (CNS) explained a significantly larger proportion of genetic variance, compared to other genic or non-genic regions, for the cognitive traits. In the annotation analysis, we divided the genome-wide SNPs into three groups; those located within ± 50 kb from the 5’ and 3’ UTR of 2,772 genes that were differentially expressed in CNS^49^; those located within the other genes except the CNS genes; and the rest of the SNPs. We partitioned the genetic variance and covariance between the four cognitive traits using a three component model fitting genomic relations matrices constructed based on the CNS, genic and non-genic SNPs.

### Software

The models and methods used in this study have been fully implemented in publicly available software, MTG2. The source code, executive binary file, manual and examples are readily available to use, and can be downloaded from https://sites.google.com/site/honglee0707/mtg2.

## Electronic supplementary material

Supplementary File
