# Supplementary material for: Genotype-environment interaction on human cognitive function conditioned on the status of breastfeeding and maternal smoking around birth
Source: Sci Rep. 2017 Jul 20;7:6087. doi: 10.1038/s41598-017-06214-y (PMC5519601; doi:10.1038/s41598-017-06214-y)
Supplement: Supplementary file 1 — Supplementary File [file 41598_2017_6214_MOESM1_ESM.pdf]

Supplementary Data

Genotype-environment interaction on human cognitive function conditioned on the status of breastfeeding and maternal smoking around birth

S. Hong Lee, W.M. Shalanee P. Weerasinghe and Julius H.J. van der Werf

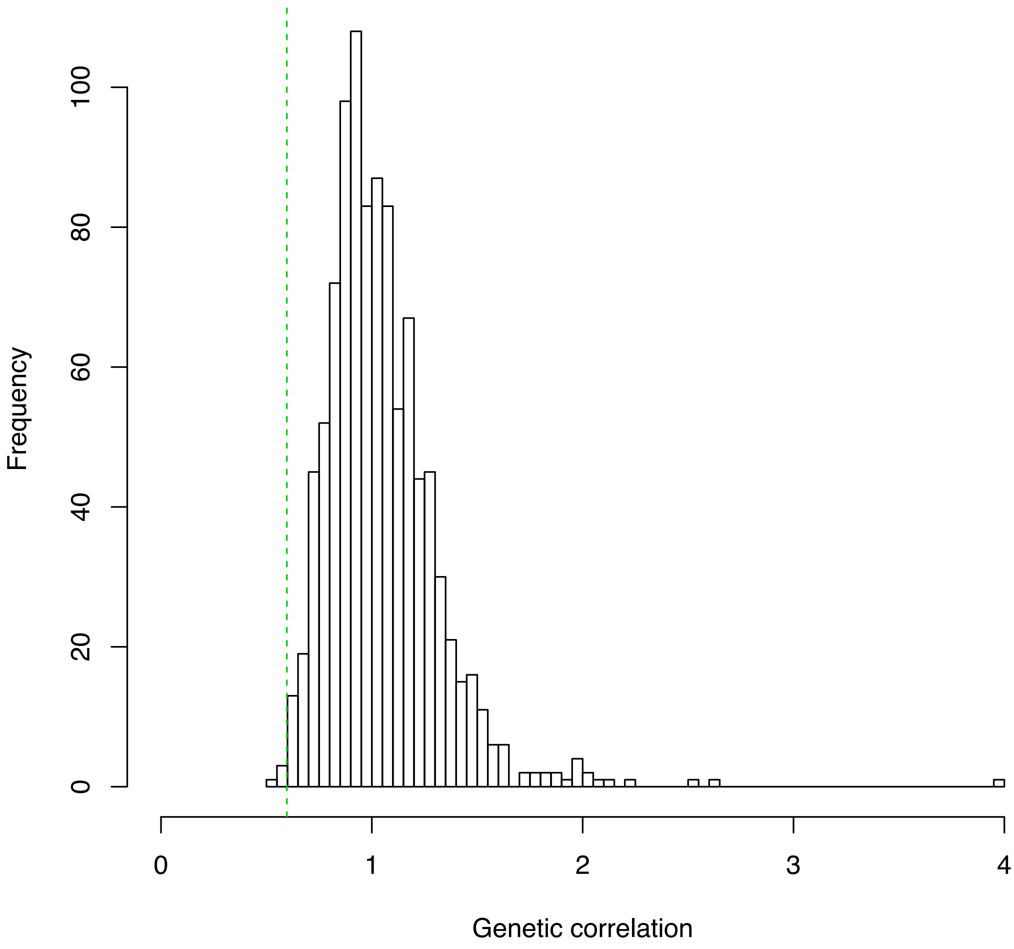

Supplementary Figure 1. The distribution of estimated genetic correlations (r_G) between NB&NS and B&NS for fluid intelligence when shuffling the environmental variable, i.e. randomly assigning NB&NS and B&NS status to the sample. The number of permutation tests was 1000. The vertical line is the original estimate (permutation p-value = 0.004).

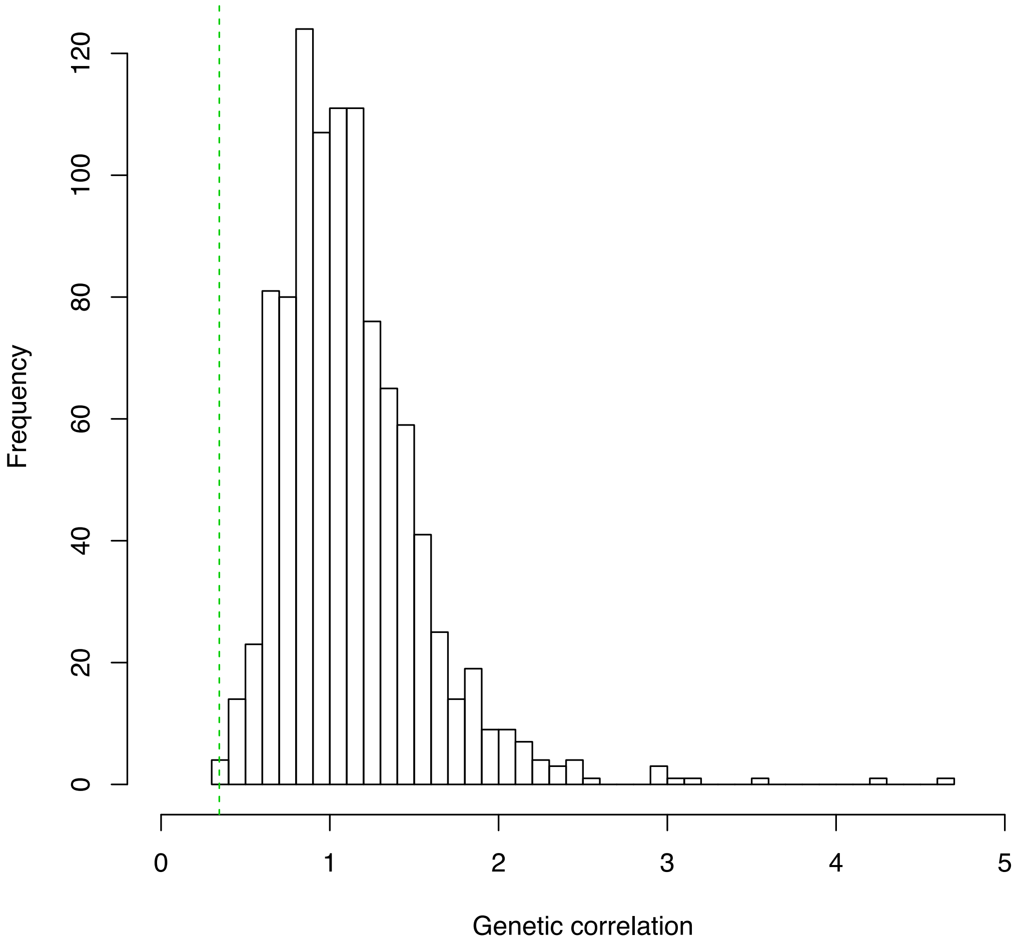

Supplementary Figure 2. The distribution of estimated genetic correlations (r_G) between NB&NS and B&S for fluid intelligence when shuffling the environmental variable, i.e. randomly assigning NB&NS and B&S status to the sample. The number of permutation tests was 1000. The vertical line is the original estimate (permutation p-value = 0.001).

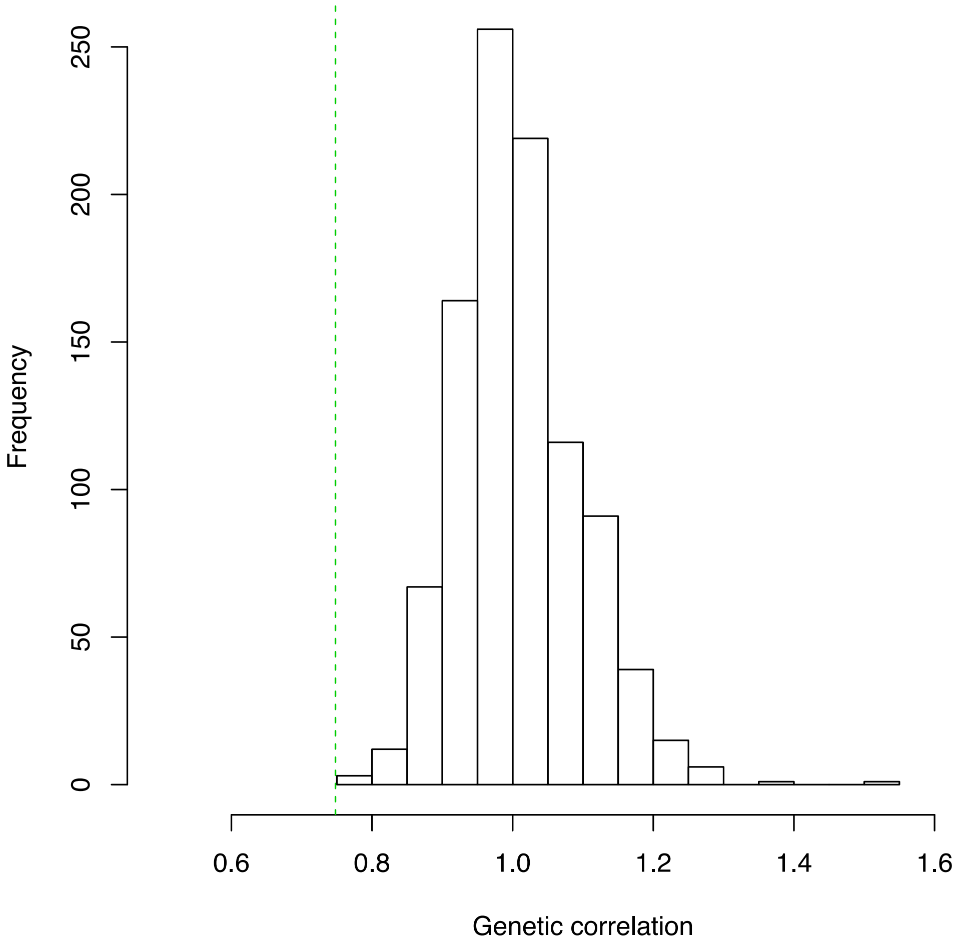

Supplementary Figure 3. The distribution of estimated genetic correlations (r_G) between NB&NS and B&NS for educational attainment when shuffling the environmental variable, i.e. randomly assigning NB&NS and B&NS status to the sample. The number of permutation tests was 1000. The vertical line is the original estimate (permutation p-value < 0.001).

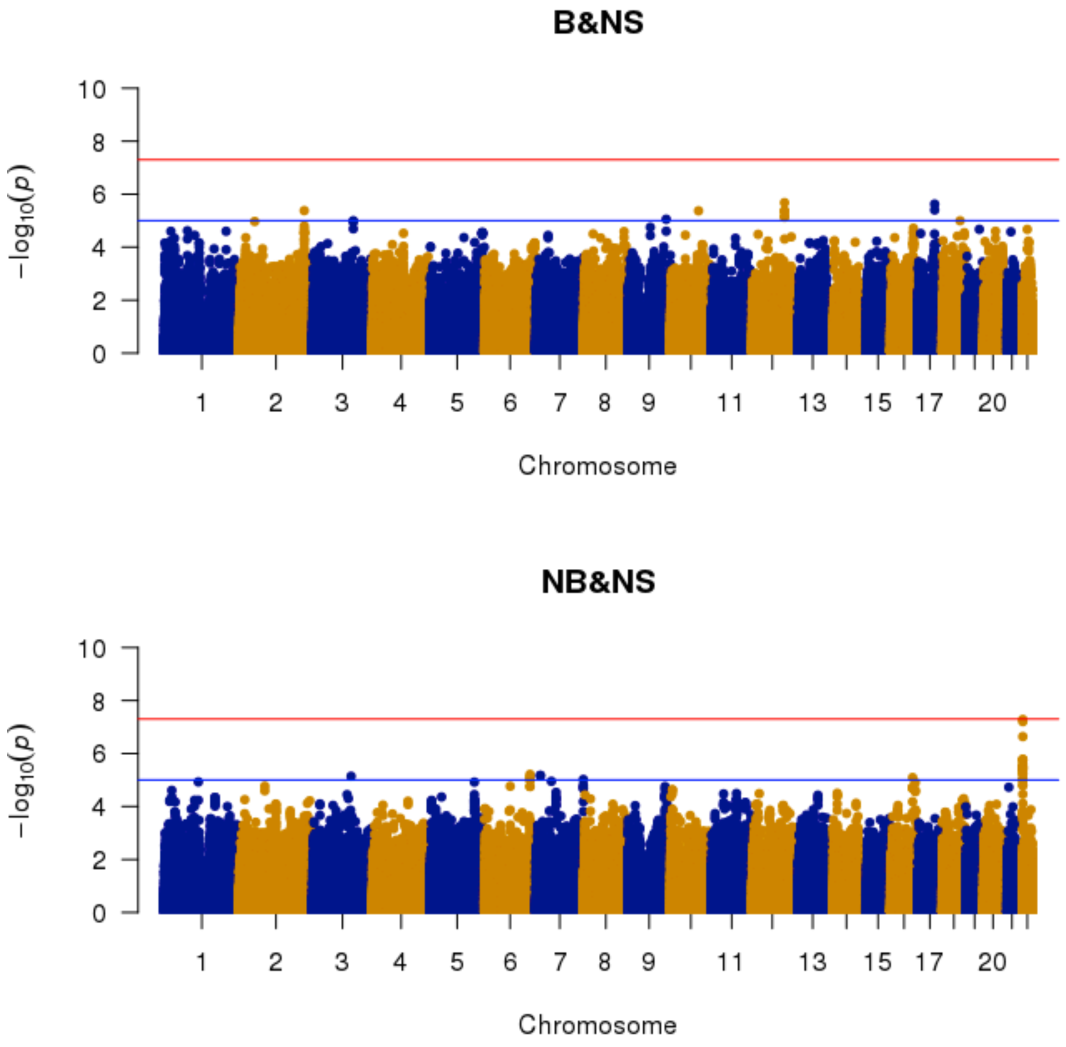

Supplementary Figure 4. Heterogeneous genome-wide significant SNP between GWAS based on B&NS and NB&NS for fluid intelligence. Genome-wide significant threshold is 5E-08.

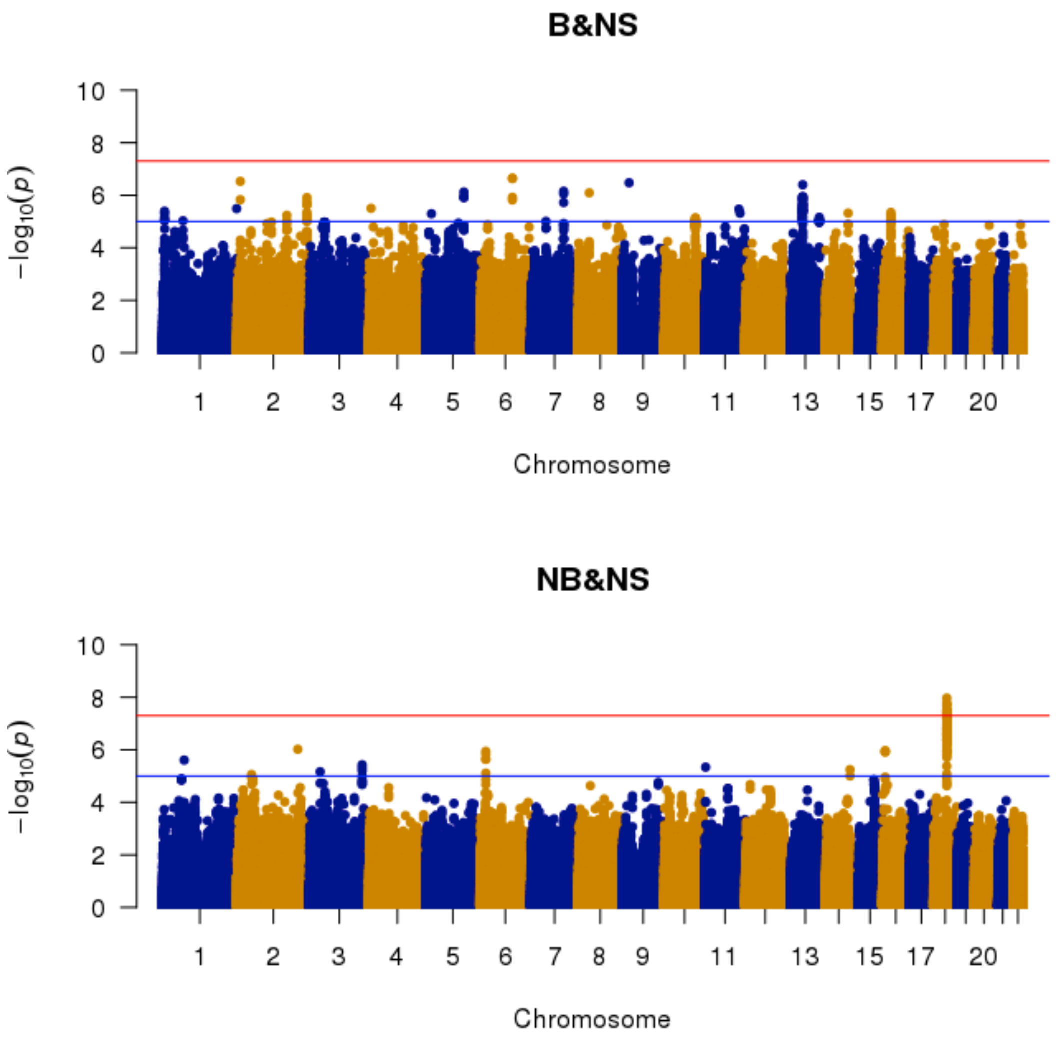

Supplementary Figure 5. Heterogeneous genome-wide significant SNP between GWAS based on B&NS and NB&NS for educational attainment. Genome-wide significant threshold is 5E-08.

A.

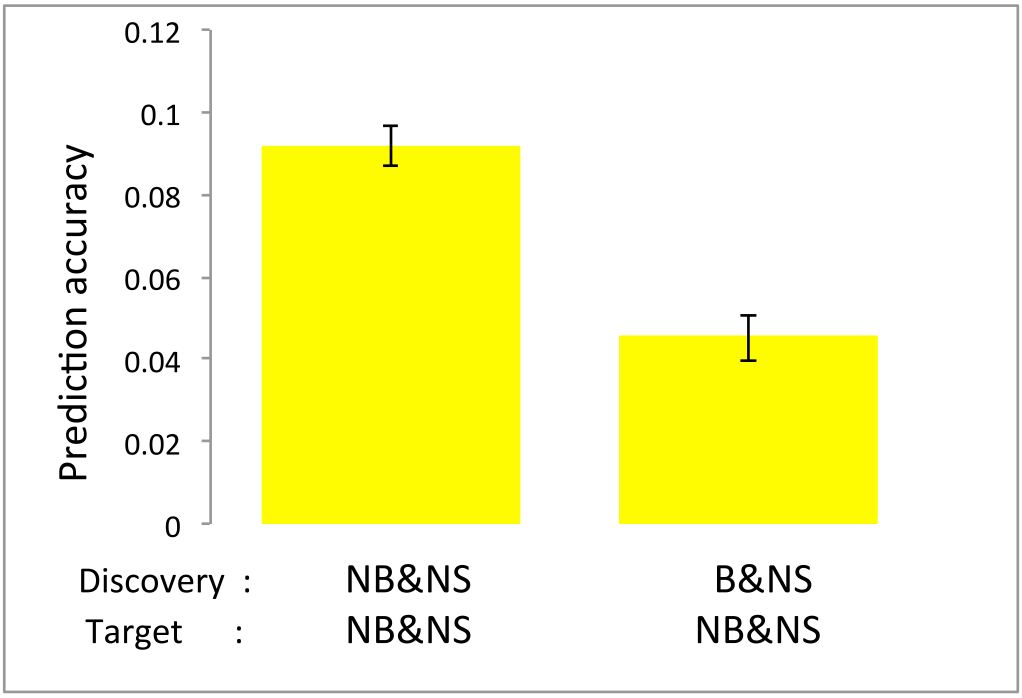

B.

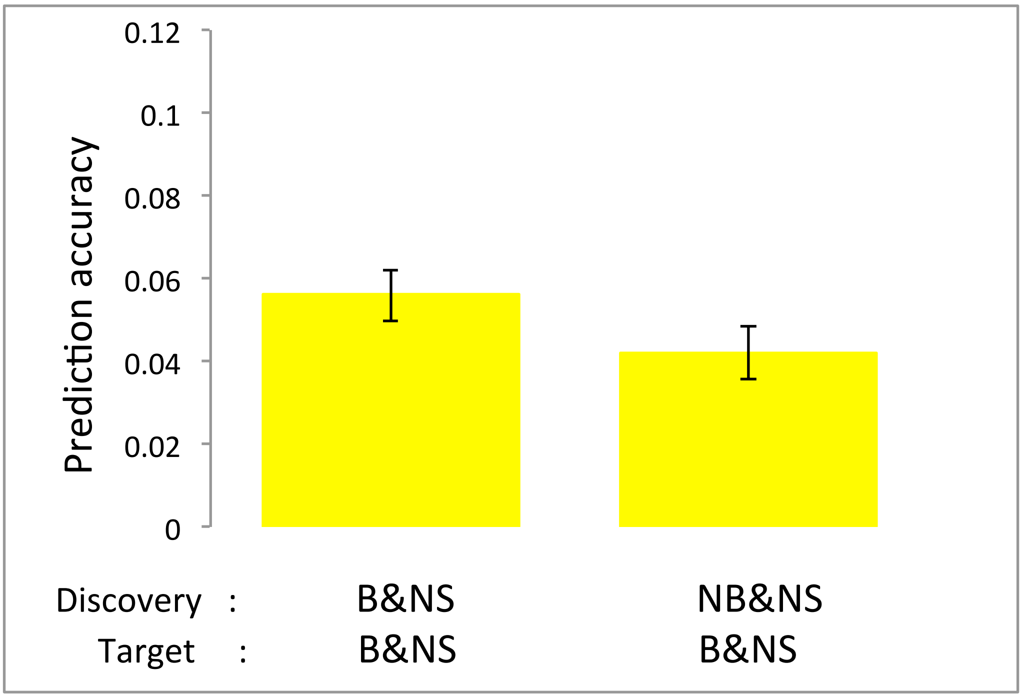

Supplementary Figure 6. Genomic prediction accuracy when using random 1000 individuals from the first group as a validation data set and random 3362 individuals from the first and second group as a discovery data set. **A**. NB&NS group was used as validation. **B**. B&NS group was used as validation. Fluid intelligence was used. The error bar is a 95% confidence interval over the 100 replicates.

A.

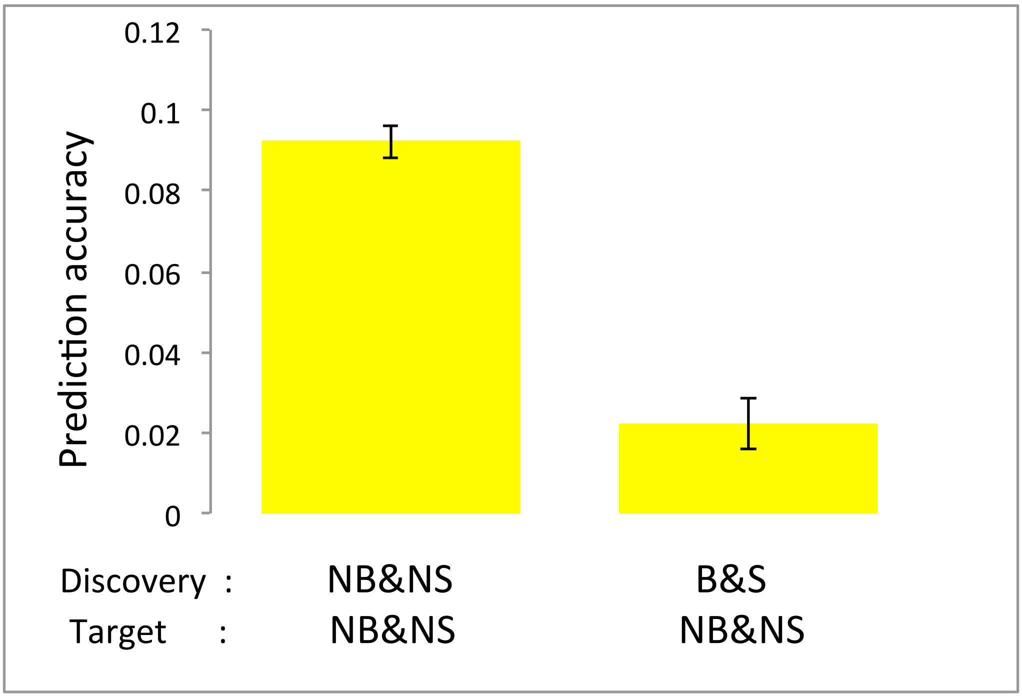

B.

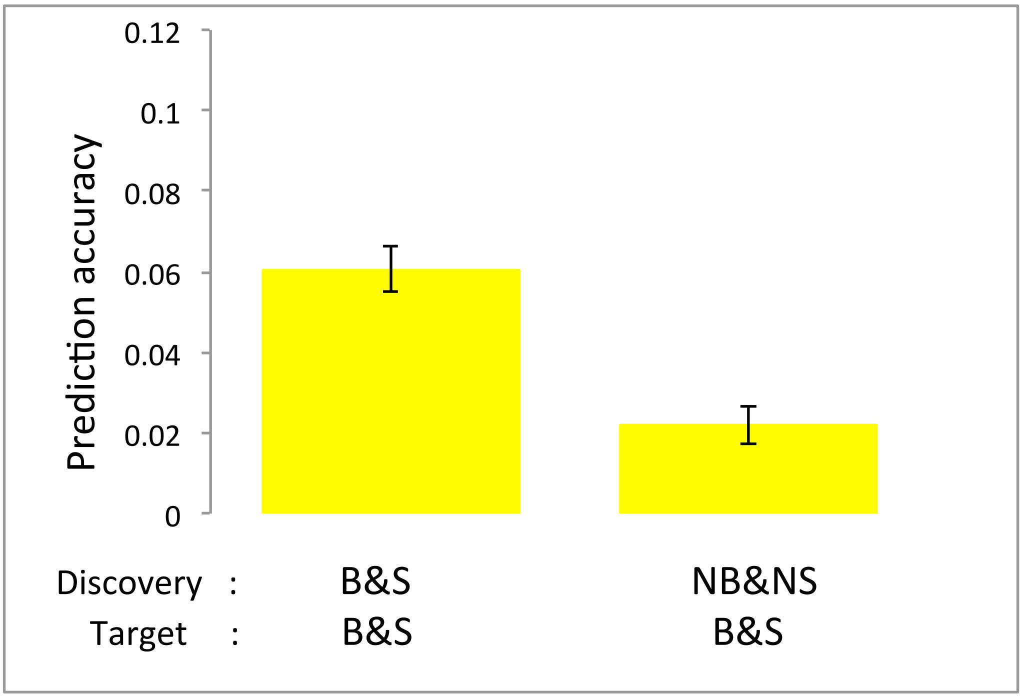

Supplementary Figure 7. Genomic prediction accuracy when using random 1000 individuals from the first group as a validation data set and random 3362 individuals from the first and second group as a discovery data set. **A**. NB&NS group was used as validation. **B**. B&S group was used as validation. Fluid intelligence was used. The error bar is a 95% confidence interval over the 100 replicates.

A.

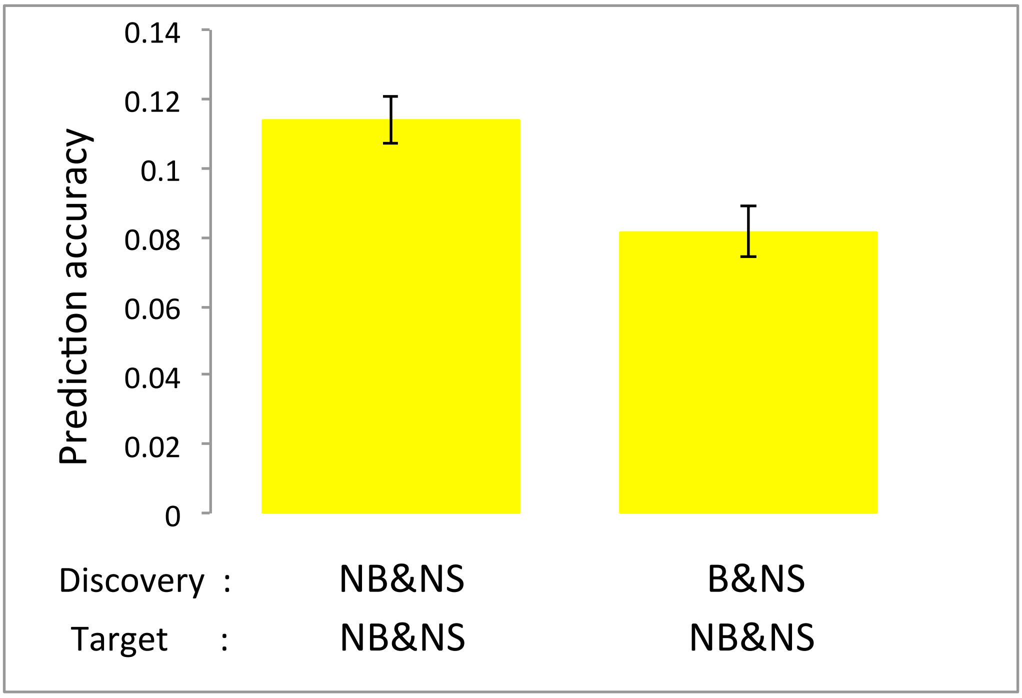

B.

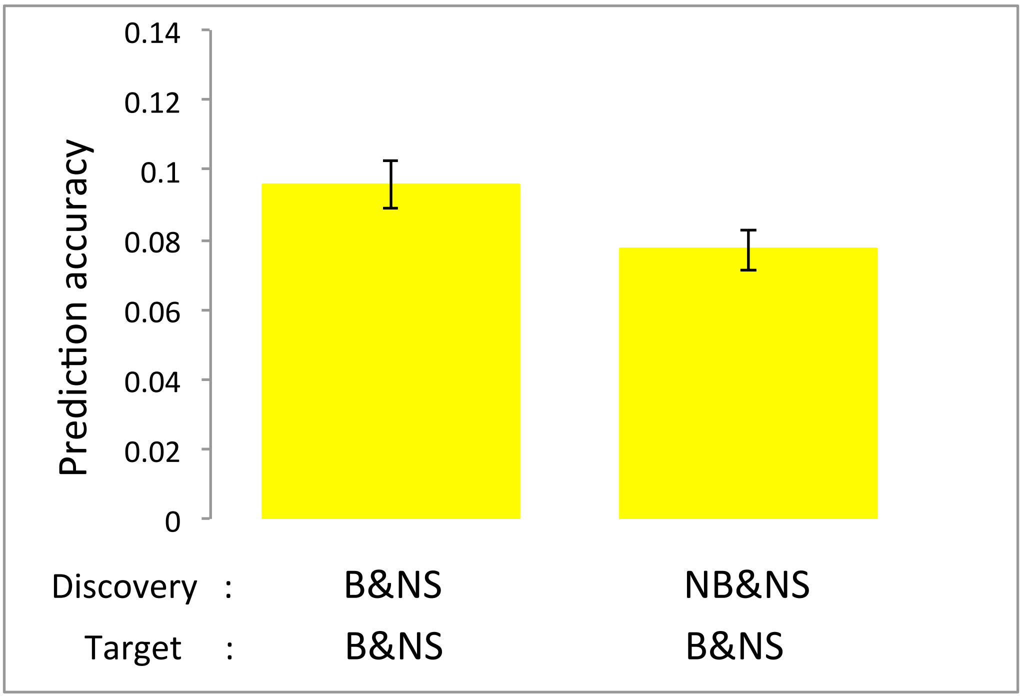

Supplementary Figure 8. Genomic prediction accuracy when using random 1000 individuals from the first group as a validation data set and random 3362 individuals from the first and second group as a discovery data set. **A**. NB&NS group was used as validation. **B**. B&NS group was used as validation. Educational attainment was used. The error bar is a 95% confidence interval over the 100 replicates.

Supplementary Table 1. The proportion of the phenotypic variance and genetic correlation between the status of breastfeeding and maternal smoking around birth for memory

	Estimate	SE	P-value	
h^2 for B&NS	0.077	0.008	8.0E-22[a]	***
h^2 for B&S	0.088	0.019	5.8E-06	***
h^2 for NB&NS	0.049	0.021	1.9E-02	*
h^2 for NB&S	0.043	0.036	2.3E-01	
r_G (B&S, B&NS)	0.770	0.143	1.1E-01[b]	
r_G (NB&NS, B&NS)	1.063	0.272	8.2E-01	
r_G (NB&NS, B&S)	1.106	0.337	7.5E-01	
r_G (NB&S, B&NS)	0.925	0.437	8.6E-01	
r_G (NB&S, B&S)	1.743	0.797	3.5E-01	
r_G (NB&S, NB&NS)	1.111	0.684	8.7E-01	

[a]testing if the estimate is different from 0. [b]testing if the estimate is different from 1.
***P-value < 0.001; **P-value < 0.01; *P-value < 0.05.

Supplementary Table 2. The proportion of the phenotypic variance and genetic correlation between the status of breastfeeding and maternal smoking around birth for reaction time

	Estimate	SE	P-value	
h^2 for B&NS	0.088	0.009	6.6E-23[a]	***
h^2 for B&S	0.063	0.020	1.2E-03	***
h^2 for NB&NS	0.072	0.023	1.6E-03	**
h^2 for NB&S	0.121	0.038	1.3E-03	**
r_G (B&S, B&NS)	1.049	0.200	8.1E-01[b]	
r_G (NB&NS, B&NS)	0.927	0.189	7.0E-01	
r_G (NB&NS, B&S)	1.010	0.304	9.7E-01	
r_G (NB&S, B&NS)	0.896	0.183	5.7E-01	
r_G (NB&S, B&S)	1.000	0.301	1.0E+00	
r_G (NB&S, NB&NS)	0.703	0.265	2.6E-01	

[a]testing if the estimate is different from 0. [b]testing if the estimate is different from 1.
***P-value < 0.001; **P-value < 0.01; *P-value < 0.05.

Supplementary Table 3. The proportion of the phenotypic variance and genetic correlation between the status of breastfeeding and maternal smoking around birth for fluid intelligence in a sex-stratified analysis

	Estimate	SE	p-value	
h^2 for $B\&NS_M$	0.241	0.053	5.6E-06[a]	***
h^2 for $B\&S_M$	0.099	0.119	4.1E-01	
h^2 for $NB\&NS_M$	0.636	0.179	3.9E-04	***
h^2 for $NB\&S_M$	0.669	0.319	3.6E-02	*
h^2 for $B\&NS_F$	0.239	0.042	1.5E-08	***
h^2 for $B\&S_F$	0.337	0.110	2.1E-03	**
h^2 for $NB\&NS_F$	0.276	0.117	1.9E-02	*
h^2 for $NB\&S_F$	0.188	0.181	3.0E-01	
r_G ($B\&NS_F$, $B\&NS_M$)	0.807	0.172	2.6E-01[b]	
r_G ($B\&S_F$, $B\&S_M$)	1.624	1.088	5.7E-01	
r_G ($NB\&NS_F$, $NB\&NS_M$)	0.817	0.307	5.5E-01	
r_G ($NB\&S_F$, $NB\&S_M$)	-0.028	0.483	3.3E-02	*

[a]testing if the estimate is different from 0

[b]testing if the estimate is different from 1; There is no evidence for sex interaction after considering a multiple testing correction.

[***]P-value < 0.001; [**]P-value < 0.01; [*]P-value < 0.05.

Supplementary Table 4. The proportion of the phenotypic variance and genetic correlation between the status of breastfeeding and maternal smoking around birth for educational attainment in a sex-stratified analysis

	Estimate	SE	p-value	
h^2 for $B\&NS_M$	0.208	0.019	3.2E-28[a]	***
h^2 for $B\&S_M$	0.136	0.040	6.1E-04	***
h^2 for $NB\&NS_M$	0.257	0.057	7.3E-06	***
h^2 for $NB\&S_M$	0.157	0.088	7.4E-02	
h^2 for $B\&NS_F$	0.187	0.015	8.6E-34	***
h^2 for $B\&S_F$	0.206	0.037	3.4E-08	***
h^2 for $NB\&NS_F$	0.217	0.036	2.2E-09	***
h^2 for $NB\&S_F$	0.224	0.061	2.1E-04	***
r_G ($B\&NS_F$, $B\&NS_M$)	0.866	0.073	6.8E-02[b]	
r_G ($B\&S_F$, $B\&S_M$)	0.949	0.218	8.2E-01	
r_G ($NB\&NS_F$, $NB\&NS_M$)	0.816	0.172	2.8E-01	
r_G ($NB\&S_F$, $NB\&S_M$)	0.659	0.342	3.2E-01	

[a]testing if the estimate is different from 0
[b]testing if the estimate is different from 1; There is no evidence for sex interaction.
***P-value < 0.001; **P-value < 0.01; *P-value < 0.05.

Supplementary Table 5. The proportion of the phenotypic variance and genetic correlation between the groups with and without breastfeeding for fluid intelligence and educational attainment

	Estimate	SE	P-value	
Fluid intelligence				
h^2 for B	0.226	0.019	4.4E-34[a]	***
h^2 for NB	0.277	0.047	3.7E-09	***
r_G (B, NB)	0.713	0.102	4.8E-03[b]	**
Educational attainment				
h^2 for B	0.177	0.007	1.4E-140[a]	***
h^2 for NB	0.192	0.015	6.4E-38	***
r_G (B, NB)	0.903	0.048	4.2E-02[b]	*

[a]testing if the estimate is different from 0. [b]testing if the estimate is different from 1. [***]P-value < 0.001; [**]P-value < 0.01; [*]P-value < 0.05.

Supplementary Table 6. The proportion of the phenotypic variance and genetic correlation between the groups with and without maternal smoking around birth for fluid intelligence and educational attainment

	Estimate	SE	P-value	
Fluid intelligence				
h^2 for NS	0.212	0.019	1.9E-28[a]	***
h^2 for S	0.234	0.041	8.5E-09	***
r_G (NS, S)	0.912	0.115	4.5E-01[b]	
Educational attainment				
h^2 for NS	0.174	0.007	2.5E-129[a]	***
h^2 for S	0.177	0.014	6.1E-37	***
r_G (NS, S)	0.967	0.050	5.1E-01[b]	

[a]testing if the estimate is different from 0. [b]testing if the estimate is different from 1.
***P-value < 0.001; **P-value < 0.01; *P-value < 0.05.

Supplementary Table 7. The number of samples for the status of breastfeeding and maternal smoking around birth when including information of average total household income in the analysis model

	Fluid intelligence	Memory	Reaction time	Educational attainment
B&NS	11736	34865	34731	34754
B&S	4748	14275	16021	14227
NB&NS	3872	12272	12216	12237
NB&S	2299	7487	7439	7456
Sum	22655	68899	68601	68674

Supplementary Table 8. The proportion of the phenotypic variance and genetic correlation between the status of breastfeeding and maternal smoking around birth for fluid intelligence when additionally adjusting phenotypes for average total household income

	Estimate	SE	P-value	
h^2 for B&NS	0.199	0.028	6.1E-13[a]	***
h^2 for B&S	0.224	0.067	8.5E-04	***
h^2 for NB&NS	0.333	0.081	4.0E-05	***
h^2 for NB&S	0.203	0.132	1.2E-01	
r_G (B&S, B&NS)	0.942	0.198	7.7E-01[b]	
r_G (NB&NS, B&NS)	0.614	0.153	1.2E-02	*
r_G (NB&NS, B&S)	0.429	0.205	5.3E-03	**
r_G (NB&S, B&NS)	1.135	0.418	7.5E-01	
r_G (NB&S, B&S)	1.043	0.465	9.3E-01	
r_G (NB&S, NB&NS)	0.905	0.412	8.2E-01	

[a]testing if the estimate is different from 0. [b]testing if the estimate is different from 1.
[***]P-value < 0.001; [**]P-value < 0.01; [*]P-value < 0.05.

Supplementary Table 9. The proportion of the phenotypic variance and genetic correlation between the status of breastfeeding and maternal smoking around birth for educational attainment when additionally adjusting phenotypes for average total household income

	Estimate	SE	P-value	
h^2 for B&NS	0.151	0.010	2.1E-49[a]	***
h^2 for B&S	0.143	0.023	4.0E-10	***
h^2 for NB&NS	0.183	0.026	2.5E-12	***
h^2 for NB&S	0.111	0.042	7.5E-03	**
r_G (B&S, B&NS)	0.867	0.097	1.7E-01[b]	
r_G (NB&NS, B&NS)	0.764	0.086	5.9E-03	**
r_G (NB&NS, B&S)	0.828	0.131	1.9E-01	
r_G (NB&S, B&NS)	1.177	0.236	4.5E-01	
r_G (NB&S, B&S)	1.029	0.258	9.1E-01	
r_G (NB&S, NB&NS)	1.036	0.255	8.9E-01	

[a]testing if the estimate is different from 0. [b]testing if the estimate is different from 1.
[***]P-value < 0.001; [**]P-value < 0.01; [*]P-value < 0.05.

Supplementary Table 10. The coefficient of determination from a regression the sex or year of birth information as a dependent variable and the 'known' and 'unknown' answer as an explanatory variable.

	Coefficient of determination (R^2)
	Sex information as dependent variable
Breastfeeding	0.0134
Maternal smoking	0.0003
	Year of birth information as dependent variable
Breastfeeding	0.0249
Maternal smoking	0.0033

Supplementary Table 11. Breastfeeding and maternal smoking rate in UK Biobank for participants born before 1950, between 1950 – 1960 and after 1960.

Breastfeeding status			
	Breastfed	Non-breastfed	Rate
< 1950	39626	10393	0.79
1950 - 1960	28499	11083	0.72
1060 <	26163	15196	0.63
Maternal smoking status			
	Smoking	Non-smoking	Rate
< 1950	17727	42911	0.29
1950 - 1960	14980	28096	0.35
1060 <	8369	18933	0.31

Supplementary Table 12. Skewness and Kurtosis of the adjusted phenotypes.

	Before inverse normal transformation		After inverse normal transformation	
	Skewness	Kurtosis	Skewness	Kurtosis
Fluid intelligence	0.148	2.87	0	2.998
Memory	4.327	48.777	0	2.999
Reaction time	1.823	11.149	0	2.999
Educational attainment	-0.139	1.777	0	2.999
